# Production of antibodies against antigens released from human pancreatic tumour xenografts.

**DOI:** 10.1038/bjc.1981.196

**Published:** 1981-09

**Authors:** A. G. Grant, D. Duke

## Abstract

Antibodies directed against the antigen released from viable tumour cells during growth have been raised by cross-immunization of immunocompetent hairy litter-mates with serum from nude mice bearing pancreatic tumour xenografts. Antisera raised against the components released from a primary pancreatic tumour xenograft (WB) or from a tumour cell-line xenograft (GER) showed a titre greater than 1:625 against cultured pancreatic tumour cells by an I125-binding assay. Five out of 14 of the hairy litter-mates immunized with serum from the same tumour (GER) produced antisera that bound more strongly to pancreatic cancer cells than to 13 other non-pancreatic cell lines (binding ratio greater than 2). Absorption of the antisera with pure CEA reduced the level of binding by 11-25% without affecting the specificity for pancreatic tumour cells. The antibody response could be completely removed by absorption with pancreatic tumour cells, whereas 50% and 18% of the activity remained after absorption with normal pancreas homogenate and a mixed non-pancreatic tumour-cell pool, respectively. Immunofluorescent staining of pancreatic tumour sections indicated that the antibody was localized on the membrane of ductular epithelial cells. Challenge of immunocompetent mice using this procedure has produced a polyspecific antiserum with signs of selectivity for the circulating antigens released from pancreatic cancer cells, and may provide a route to the production of antibody against specific tumour components.


*Br. J. Cancer* (1981) **44**, 388

### PRODUCTION OF ANTIBODIES AGAINST ANTIGENS RELEASED FROM HUMAN PANCREATIC TUMOUR XENOGRAFTS

A. G. GRANT AND D. DUKE*

*From the Department of Surgery, St George's Hospital Medical School, London SW17 0RE, and the *Department of Cancer Chemotherapy, Imperial Cancer Research Fund, Lincoln's Inn Fields, London WC2A 3PX*

Received 22 January 1981 Accepted 7 May 1981

**Summary.—Antibodies directed against the antigens released from viable tumour cells during growth have been raised by cross-immunization of immunocompetent hairy litter-mates with serum from nude mice bearing pancreatic tumour xenografts. Antisera raised against the components released from a primary pancreatic tumour xenograft (WB) or from a tumour cell-line xenograft (GER) showed a titre >1:625 against cultured pancreatic tumour cells by an $I^{125}$-binding assay. Five out of 14 of the hairy litter-mates immunized with serum from the same tumour (GER) produced antisera that bound more strongly to pancreatic cancer cells than to 13 other non-pancreatic cell lines (binding ratio >2). Absorption of the antisera with pure CEA reduced the level of binding by 11–25% without affecting the specificity for pancreatic tumour cells. The antibody response could be completely removed by absorption with pancreatic tumour cells, whereas 50% and 18% of the activity remained after absorption with normal pancreas homogenate and a mixed non-pancreatic tumour-cell pool, respectively. Immunofluorescent staining of pancreatic tumour sections indicated that the antibody was localized on the membrane of ductular epithelial cells. Challenge of immunocompetent mice using this procedure has produced a poly-specific antiserum with signs of selectivity for the circulating antigens released from pancreatic cancer cells, and may provide a route to the production of antibody against specific tumour components.**

CANCER CELLS may evade immunological recognition by shedding their surface antigens (Alexander, 1974; Baldwin *et al.*, 1974). These antigens are glycoproteins or whole plasma membrane fragments (De Broe *et al.*, 1977) which circulate in a free state or complex with host immunoglobulins (Sjögren *et al.*, 1971; Bowen *et al.*, 1975). Although a number of groups have demonstrated the release of human and animal cell-surface proteins into the serum of tumour-bearing animals (Jamasbi *et al.*, 1978; Nordquist *et al.*, 1978; Rao & Bonavida, 1977; Primus *et al.*, 1976) a study of this process is complicated by the host's immune system.

The availability of human tumour xenografts grown in nude mice provides a model in which human proteins are released into the blood stream of an immune-deficient animal. The aim of this study was to ascertain whether an antibody response against these circulating human tumour antigens could be achieved by injection of serum from tumour-bearing animals into immunocompetent hairy litter-mates. Using this immunization regime it should be possible to produce a polyspecific antiserum recognising both tumour and normal components that are shed from the cell surface. The possibility of selecting antibodies to specific circulating tumour components by eventually fusing the spleen cells of these animals with a myeloma to produce monoclonal antibodies should be applicable to all solid tumours, and may have considerable clinical application.

### MATERIALS AND METHODS

*Animals.*—Four–6-week-old female congenitally athymic (*nu/nu*) mice and their hairy litter-mates were bred at the Imperial Cancer Research Fund (ICRF) Laboratories. Nude mice were bred and maintained in sterile conditions in a negative-pressure isolator. Hairy litter mates (*nu*/+) were maintained in conventional conditions.

*Cell lines.*—Human pancreatic exocrine adenocarcinoma cells (GER) and a normal human fibroblast cell line derived from the same tumour tissue (GF) were cultured and harvested as previously described (Grant *et al.*, 1979). Human foetal pancreatic fibroblasts (FB) derived from a 9-week-old embryo (Tissue Bank, Royal Marsden Hospital, London) and a fibroblast cell line from a second pancreatic tumour (HF) were established in a similar way.

Lymphoblastoid and myeloid cell lines HSB2 (Adams *et al.*, 1968) and HL60 (Collins *et al.*, 1979), and a breast carcinoma line MDA-157 (Young *et al.*, 1974) were obtained from P. Beverley, ICRF, London. Human urinary-bladder carcinoma cell lines SCaBer (O'Toole *et al.*, 1976), TCC-sup (Nayak *et al.*, 1977), J82 (O'Toole *et al.*, 1978), T-24 (Bubenik *et al.*, 1973), a line of non-malignant bladder epithelium, HCV-29 (derived by J. Fogh), and a colon-carcinoma cell line HT-29 (Fogh & Trempe, 1975) were kindly provided by C. O'Toole, London Hospital Medical College. Peripheral-blood lymphocytes (PBL) were isolated from the blood of patients with pancreatic cancer and from normal healthy donors. Twenty ml of blood was collected in 1 ml 2·7% EDTA in phosphate-buffered saline (PBS) or was defibrinated on glass beads to prevent clotting. The cells were isolated by Ficoll-paque (Pharmacia) separation. Lymphocytes from one pancreatic cancer patient were transformed with Epstein–Barr (EBV) virus by C. O'Toole using the method of Miller & Lipman (1973).

All cell lines were maintained in Ham's F12 medium supplemented with 10% foetal bovine serum and harvested with 0·02% EDTA in Ca- and Mg-free Eearle's (Flow Laboratories).

*Human tumour xenografts.*—Primary pancreatic tumour tissue (WB) or a pancreatic adenocarcinoma cell line (GER) were used to establish solid human tumour xenografts in nude mice as previously described (Grant *et al.*, 1979). A murine tumour (MP) was induced in nude mice with polyoma virus. To establish cell lines from the xenografts, tissue was minced with crossed scalpels and dispersed into 25cm$^2$ flasks with 3 ml of medium. Tumour cells formed colonies of epithelial cells, which were passaged after 3–4 weeks. Human or murine tumour origin was confirmed by chromosome analysis (Schwartzacher & Wolf, 1974).

*Immunization of hairy litter-mates with serum from tumour-bearing nude mice.*—Serum was collected by cardiac puncture from 20 tumour-bearing nude mice when the tumours measured 1·25 cm$^2$. Mice in 4 groups (GER-A, B, D; WB) of 6 hairy litter-mates received 0·4 ml of serum, emulsified in complete Freund's adjuvant (1:1 v/v), which was divided between 4 sites and given s.c. on Day 0. Two similar injections were given at 14 and 28 days, and followed by 0·4 ml serum i.p. at 42 days. Half a ml of blood was collected from the tail vein 6 days after the final immunization, to confirm the presence of antibody, and the mice bled out 4 days after the test bleed. Two groups of 6 hairy litter-mates received normal nude mouse serum with the same immunization regime (Groups F & C).

Antibodies directed against normal human lymphocytes were raised in 6 CBA mice by 3 injections of $1{\cdot}5\times10^7$ PBL at 0, 7 and 21 days. This antiserum was used as a positive control (MHL).

*Alpha-foetoprotein (AFP) and carcino-embryonic antigen (CEA) estimations.*—Tumour-bearing mouse serum AFP and CEA levels were measured by the Protein Reference Unit, Putney Hospital, and the Department of Clinical Immunology, Charing Cross Hospital, respectively, using an adaptation of the AFP radioimmunoassay method of Nishi & Hirai (1973), and the CEA double-antibody radioimmunoassay of Laurence *et al.* (1972).

*Immunofluorescence studies.*—Suspensions of viable cells ($0{\cdot}5\text{–}1\times10^6$ cells/well) were incubated with 50–100 $\mu$l hairy litter-mate serum raised against tumour-bearing nude mouse serum or normal nude mouse serum. Antisera were diluted 1:5–1:20 in PBS for use in the assay. Mouse anti-human lymphocyte serum (MHL), normal nude mouse serum (NS) and serum obtained directly from tumour-bearing nude mice (NMG, NMW) were also included in the study.

Cells were incubated at 4°C for 30 min, washed ×3 with PBS, resuspended in 50 $\mu$l PBS and labelled with 5–10 $\mu$l TRIC-conjugated anti-mouse IgG (Nordic Immunological Laboratories, Maidenhead) for 30 min at 4°C. Cells were washed a further ×3 in PBS, mounted in PBS/glycerol (1:1 v/v) and examined with a Zeiss photomicroscope with epi-fluorescent illumination.

Primary tumour tissue or xenografts were embedded in OCT compound (Lab-Tek Products, Miles Laboratories), snap-frozen in isopentane/liquid $N_2$ and stored at −70°C. Five-$\mu$m sections were cut on a cryostat. Fluorescence was detected using Coons' sandwich technique (Coons *et al.*, 1955).

*Antibody-binding assay.*—Binding of $I^{125}$ anti-mouse immunoglobulin to target cells was performed essentially as described by Stern *et al.* (1978). Suspensions of viable cells ($2–5 \times 10^5$ cells/well) were incubated with 50 $\mu$l antiserum (1:5–1:625 dilution) followed by 20 $\mu$l of $I^{125}$ sheep antimouse Ig (30,000 ct/min) (a gift from P. Beverley). The ratios of counts bound with antiserum against tumour-bearing serum to counts bound with antiserum against normal nude mouse serum, or of counts bound to pancreatic target cells *vs* counts bound to other target cells, were calculated. A ratio of 2 or more was considered to be significant binding.

For CEA absorption 50 $\mu$l of 1:40 dilution of antisera was absorbed with 0·03–4 $\mu$g CEA (a gift from J. Westwood, Institute of Cancer Research, Sutton, Surrey) or 0·05–0·5 $\mu$g AFP (Dako-Immunoglobulins, Denmark) for 15–60 min at room temperature. Antigen–antibody complexes were removed with Staphylococcal protein-A antibody absorbent (Porton Down) according to the method of Kessler (1975) before adding the cells. Antisera were also absorbed with $5 \times 10^5–2 \times 10^7$ viable cell suspensions or 0·3–3·4 mg normal pancreas homogenate insolubilized in 2·5% gluteraldehyde/PBS, for 60 min at room temperature, before the antibody binding assay. Protein content was estimated according to the Lowry method.

### RESULTS

### *Screening of antisera from hairy litter-mates for antibody against tumour cells*

All hairy litter-mates immunized with sera from nude mice bearing xenografts derived from a pancreatic tumour cell line (GER) or primary tumour tissue (WB) produced antibodies which bound to pancreatic tumour cells, as shown by indirect immunofluorescence (Fig. 1). No antibodies were detected in the sera of

FIG. 1.—Screening antisera for antibody against pancreatic tumour cells. Individual hairy litter-mates immunized with serum from nude mice bearing either GER or WB tumours were tested for antibodies binding to cultured pancreatic (GER) or urinary bladder (TCC-sup) tumour cells. $5 \times 10^5$ cells were incubated with 50 $\mu$l of a 1:20 (fluorescence) or 1:40 ($I^{125}$ binding) dilution of antisera followed by TRIC or $I^{125}$ anti-mouse Ig as described in Methods. MHL: mouse anti-human lymphocytes (positive control) Antisera A-D: mice immunized with GER tumour; WB: mice immunized with WB tumour; F; mice immunized with normal nude mouse sera; NMG: immunizing sera from mouse bearing GER tumour; NW: immunizing sera from mouse bearing WB tumour.

FIG. 2.—Serial dilutions of antiserum tested for binding to pancreatic, tumour cells. $5 \times 10^5$ cells were incubated with 50 $\mu$l of 1:5 to 1:625 dilutions of anti-tumour-bearing serum DNM (●——●) or anti-normal nude mouse serum FIR (○——○) followed by 20 $\mu$l $I^{125}$ anti-mouse Ig (30,000 ct/min) as described in Methods.

hairy litter-mates immunized with normal nude mouse serum (FIR, FIL, F2L) or in the immunizing serum from tumour-bearing nude mice (NMG, NMW). Using the $I^{125}$ antibody-binding assay the binding ratios between antibodies raised against tumour-bearing (GER and WB) and normal nude mouse serum were between 2 and 3 at dilutions up to 1:625 when tested on pancreatic cancer cells (Fig. 2). This assay was also able to identify quantitative differences in the antibody response of individual hairy litter-mates to sera from the same pancreatic tumour (GER) which were not evident by immunofluorescence (Fig. 1). There was no difference in the amount of antibody binding to pancreatic tumour cells cultured from primary tissue and those which had been re-established as a cell line from the xenograft.

*Specificity of the antisera for pancreatic tumour cells*

Only 5/14 hairy litter-mates immunized with tumour-bearing sera (GER) produced antibodies which were selective for pancreatic tumour cells (binding ratio $>2$) when tested against 2 human urinary bladder tumour cell lines T24 and TCC-sup. The remainder showed higher binding to pancreatic tumour cells, but the binding ratios were not $>2$. The results of screening the sera against TCC-sup are shown in Fig. 1; T24 gave essentially the same binding ratios.

Screening of one of the more specific antisera (DNM), and one of the remainder (ANM), against a panel of 14 different cell lines and PBL is shown in Fig. 3. There was considerably greater binding of the antiserum DNM to pancreatic tumour cells than to the other cell lines. Only the fibroblast cell line derived from the same pancreatic tumour (GF) and a colonic carcinoma (HT-29) had binding ratios $<2$. Although ANM showed the highest response to pancreatic tumuor cells, it was also bound more strongly to the other tumour cell lines. All cell lines responded very weakly to the serum of hairy litter-mates immunized with normal nude mouse serum (FIR; Fig. 3). In contrast, antiserum against human lymphocytes (MHL) showed a high level of binding; only the murine tumour (MP) did not react with this antiserum.

*Presence of antibodies to CEA and AFP*

There was $<25$ $\mu$g/l of AFP in the serum of mice bearing a large tumour load. Absorption of antiserum (DNM) with 0·05–0·5 $\mu$g of AFP reduced binding of the antiserum to pancreatic tumour cells by less than 10% at the highest concentration of antigen. CEA was present in the serum of tumour-bearing animals at concentrations up to 100 $\mu$g/l, but was only poorly detected at the cell surface when tested with anti-CEA (Dako Products, Denmark) by indirect immunofluorescence. Absorption of antiserum (DNM) with 0·03–4 $\mu$g CEA reduced antibody binding maximally by 30% after 15 min at room temperature, at concentrations of CEA greater than 0·8 $\mu$g.

As can be seen in Fig. 4, the amount of antiserum binding to GER, TCC, J82 and HT-29 cells was reduced by 11–24%

FIG. 3.—Binding of antisera to tumour and normal cells. $5 \times 10^5$ cells were incubated with 50$\mu$l of 1 :40 dilution of anti-tumour-bearing mouse serum (ANM, DNM), anti-normal nude mouse serum (FIR) and mouse anti-human lymphocytes (MHL) followed by 15–20$\mu$l $I^{125}$ anti-mouse Ig (30,000 ct/min). Cell lines are described in methods. N.D.—not determined. Results ($\pm$s.e., 3 expts) are expressed as $I^{125}$–Ig binding per $10^5$ cells, and as a binding ratio of counts bound to pancreatic tumour cells over counts bound to other target cells.

following CEA absorption, so that the binding ratios were maintained and DNM antisera still retained its specificity for pancreatic tumour cells. Absorption with CEA did not affect the binding of MHL to either GER, TCC, J82 or HT-29.

*Absorption of antisera with normal pancreas and a mixed tumour-cell pool*

Five $\times 10^6$ viable pancreatic tumour cells (equivalent to 1·2 mg protein) completely removed the antibody response from 50 $\mu$l of 1 : 50 dilution of antiserum after 1 h incubation at room temperature. After absorption with up to $2 \times 10^7$ cells (5 mg protein) from a mixed pool of equivalent numbers of J82, TCC, MDA, HT29 tumour cells, 18% of the activity binding to pancreatic tumour cells remained. 50% binding activity remained after absorption with 3·4 mg of a glutaraldehyde-insolubilized normal-pancreas homogenate, and the activity was reduced to 16% after sequential absorption with normal pancreas and a mixed cell pool.

*Localization of the antisera in frozen tissue sections*

Positive immunofluorescent staining was seen in sections from all primary pancreatic tumours and tumour xenografts with dilutions of CEA-absorbed antiserum up to 1 :100. Areas of strong staining were located around the malignant ductular epithelial cells, and appeared to reflect the degree of morphological differentiation of the tumour; a highly anaplastic poorly differentiated cell population (TUR) showing very little staining, whereas a well differentiated tumour (WB) was strongly positive. Staining of normal and foetal pancreas was found in ductular-cell areas. All sections were negative with hairy anti-normal nude mouse serum and gave a general positive fluorescence with MHL.

FIG. 4.—Absorption of antisera with CEA. $5 \times 10^5$ cells were incubated with 50 $\mu$l 1:40 dilution of anti-tumour-bearing mouse serum (DNM, ANM) and mouse anti-human lymphocytes (MHL). Half of the antisera were absorbed with 0·8 $\mu$g CEA for 30 min before incubation. Results are the means of 3 experiments.

DISCUSSION

Cross-immunization of immunocompetent hairy litter-mates with serum from pancreatic tumour-bearing immunodeficient nude mice has led to the production of antibodies directed against the components released from viable human tumour cells during growth. Nude mice have a normal $\beta$-lymphocyte complement (Sprent & Miller, 1972) but we were unable to detect free antibodies against pancreatic tumour cells in the sera of these tumour-bearing animals, suggesting that antigens were produced in excess of antibody and that the hairy litter-mates were challenged with both antigen and antigen–antibody complexes.

The presence of a high titre of antibodies to pancreatic tumour cells in the polyspecific antiserum was demonstrated by screening against a panel of tumour and normal diploid cell lines. This showed that the ability of immunocompetent mice to produce antibodies against human cell components varied considerably. Only 36% of the hairy litter-mates produced a high titre of antibody to pancreatic tumour cells. Since the animals all received the same pooled serum from tumour-bearing mice this is unlikely to be due to differences in serum protein composition and probably reflects the mixed origins of the immunocompetent mouse population. Preliminary studies using matched nude and hairy litter-mates (WB) might have overcome this problem, but were not successful because of the small number of animals available. These hairy litter-mates also had a nu/+ background, and a higher antibody titre might have been achieved with +/+ animals.

A mixed response to the polyspecific antiserum was shown by all the cell lines tested, suggesting that antibodies were also raised against common tumour-cell markers (CEA, epithelial-cell markers) and normal cell-surface components that had been shed from the pancreatic tumour cells. Release of these and other surface components may be a normal function of the cell, or may arise from cellular degradation. We had previously reported that pancreatic tumour cells express HLA and $\beta_2$ on their cell surface (Pahlman *et al.*, 1979) and this probably explains the high level of antibody binding to a fibroblast cell line (GF) derived from the same patient from whom the tumour cell originated. Very little CEA was detected on the pancreatic tumour cell surface but it was present in the serum of tumour-bearing animals which agreed with other studies (Kim *et al.*, 1976). However, the antibody against CEA could be removed from the sera of immunocompetent mice by adsorption with pure CEA without any loss of specificity against pancreatic tumour cells. Binding to a CEA-producing colonic carcinoma cell line (Von Kleist *et al.*, 1975) remained consistently high after CEA adsorption, suggesting that these two gastrointestinally-derived cell lines shared a number of cell-surface components. AFP was not detected in the sera of tumour-bearing animals, and adsorption with pure antigen did not affect the antibody response.

Immunofluorescent staining of tissue sections showed that the predominantly antibody response was localized around the membrane of ductular epithelial cells in tumour tissue as well as foetal and normal pancreas. However, the antibodies did not appear to be primarily directed against epithelial cells, since the 2 epithelial tumour-cell lines (SCaBer and MDA) showed only 18% and 19% respectively of the binding level found for pancreatic-cancer cells. Similarly the low level of binding to murine polyoma-virus-transformed cells confirmed that the antibodies specifically identified human tumour components.

Absorption of the antisera with a mixed tumour-cell pool and normal pancreas homogenate did not completely abolish the antibody response against pancreatic tumour cells, suggesting the presence of a pancreatic tumour-specific antigen. Although some of the antibody response is likely to be directed against pancreatic antigens that have already been isolated (Schultz & Yunis, 1979 Gelder *et al.*, 1978) it is possible that antibodies may be raised against cell-surface components not normally present in sufficient quantities to be immunogenic. Thus, identification of minor, but possibly important, tumour components might be achieved using this technique, since they will be continually released into the serum during tumour growth. Another of the advantages of this cross-immunization technique is that it offers a novel way of subsequently producing monoclonal antibodies directed against specific antigens released from the tumour cell. Spleens from immunized hairy litter-mates are currently being hybridized with myeloma cells and, since the method permits the exploitation of qualitative and quantitative differences in the recognition of human tumour components by individual immunocompetent mice, it is hoped that the resulting monoclonal antibodies will identify a variety of tumour cell-surface components.

We are most grateful to Professor John Hermon-Taylor, Professor Kurt Hellmann, Dr Peter Beverley and Dr Carol O'Toole for their valuable encouragement and support. AFP and CEA estimations were kindly provided by Dr Hilary Orr, Protein Reference Unit, Putney Hospital, and Dr Hugh Mitchell, Department of Clinical Immunology, Charing Cross Hospital. We thank Dr J. Westwood for the gift of CEA and the Tissue Bank for their help with providing foetal tissue. This work was funded in part by a grant from the Cancer Research Campaign.

### REFERENCES

ADAMS, R. A., FLOWERS, A. & DAVIS, B. J. (1968) Direct implantation and serial transplantation of human acute lymphoblastic leukaemias in hamsters, SB-2. *Cancer Res.*, **28**, 1121.

ALEXANDER, P. (1974) Escape from immune destruction by the host through shedding of surface antigens: Is this a characteristic shared by malignant and embryonic cells? *Cancer Res.*, **34**, 2077.

BALDWIN, R. W., EMBLETON, M. J., PRICE, M. R. & ROBINS, A. (1974) Immunity in the tumor-bearing host and its modification by serum factors. *Cancer*, **34**, 1452.

BOWEN, J. G., ROBINS, R. A. & BALDWIN, R. W. (1975) Serum factors modifying cell mediated immunity to rat hepatoma D23 correlated with tumour growth. *Int. J. Cancer*, **15**, 640.

BUBENIK, J., BARESOVA, M., VIKLICKY, C., JABOUBKOVA, J., SAINEROVA, H. & DONNER, J. (1973) Established cell line of urinary bladder carcinoma (T24) containing tumor-specific antigens. *Int. J. Cancer*, **11**, 765.

COLLINS, S. J., GALLO, R. C. & GALLAGHER, R. E. (1977) Continuous growth and differentiation of human myeloid leukaemic cells in suspension culture. *Nature*, **270**, 347.

COONS, A. H., LEDUC, E. H. & CONNOLLY, J. M. (1955) Studies on antibody production. *J. Exp. Med.*, **162**, 49.

DE BROE, M. A., WIEME, R. J., LOGGHE, G. N. & ROELS, F. (1977) Spontaneous shedding of plasma membrane fragments by human cells *in vivo* and *in vitro*. *Clin. Chim. Acta*, **81**, 237.

FOGH, J. & TREMPE, G. (1975) New human tumor cell lines. In *Human Tumour Cells* in Vitro. Ed. Fogh. New York: Plenum Press. p. 115.

GELDER, F. B., REESE, C. J., MOOSSA, A. R., HALL, T. & HUNTER, R. (1978) Purification, partial characterization, and clinical evaluation of a pancreatic oncofetal antigen. *Cancer Res.*, **38**, 313.

GRANT, A. G., DUKE, D. & HERMON-TAYLOR, J. (1979) Establishment and characterization of primary human pancreatic carcinoma in continuous cell culture and in nude mice. *Br. J. Cancer*, **39**, 143.

JAMASBI, R. J., NETTESHEIM, P. & KENNEL, S. J. (1978) Detection of circulating tumor antigens in mice carrying a highly metastatic pulmonary squamous-cell carcinoma. *Int. J. Cancer*, **21**, 387.

KESSLER, S. W. (1975) Rapid isolation of antigens from cells with a staphylococcal protein A-antibody adsorbent: Parameters of the interaction of antibody–antigen complexes with protein A. *J. Immunol.*, **115**, 1617.

KIM, D. K., KAKITA, A., CUBILLA, A., FLEISHER, M. & FORTNER, J. G. (1976) Unique features of serially transplanted human pancreatic cancer in nude mice. *Surg. Forum*, **27**, 142.

LAURENCE, D. J. R., STEVENS, U., BETTELHEIM, R. & 6 others (1972) Evaluation of the role of plasma carcinoembryonic antigen (CEA) in the diagnosis of gastrointestinal, mammary and bronchial carcinoma. *Br. Med. J.*, **3**, 605.

MILLER, G. & LIPMAN, M. (1973) Release of infectious Epstein–Barr virus by transformed marmoset leukocytes. *Proc. Natl Acad. Sci. U.S.A.*, **70**, 190.

NAYAK, S. K., O'TOOLE, C. & PRICE, Z. H. (1977) A cell line from an anaplastic transitional cell carcinoma of human urinary bladder. *Br. J. Cancer*, **35**, 142.

NISHI, S. & HIRAI, H. (1973) Radioimmunoassay of α-fetoprotein in hepatoma, other liver diseases, and pregnancy. *GANN*, **14**, 79.

NORDQUIST, R. E., ANGLIN, J. H. & LERNER, M. P. (1978) Antigen shedding by human breast-cancer cells *in vitro* and *in vivo*. *Br. J. Cancer*, **37**, 776.

O'TOOLE, C., NAYAK, S., PRICE, Z., GILBERT, W. H. & WAISMAN, J. (1976) A cell line (SCaBER) derived from squamous cell carcinoma of the human urinary bladder. *Int. J. Cancer*, **17**, 707.

O'TOOLE, C., PRICE, Z. H., OHNUKI, Y. & UNSGAARD, B. (1978) Ultrastructure, karyology and immunology of a cell line originated from a human transitional-cell carcinoma. *Br. J. Cancer*, **38**, 64.

PAHLMAN, S., LJUNGSTEDT-PAHLMAN, I., SANDERSON, A., WARD, P. J., GRANT, A. G. & HERMON-TAYLOR, J. (1979) Isolation of plasma-membrane components from cultured human pancreatic cancer cells by immuno-affinity chromatography of anti-$\beta_2$M sepharose 6MB. *Br. J. Cancer*, **40**, 701.

PRIMUS, F. J., WANG, R. H., COHEN, E., HANSEN, H. J. & GOLDENBERG, D. M. (1976) Antibody to carcinoembryonic antigen in hamsters bearing GW-39 human tumours. *Cancer Res.*, **36**, 2176.

RAO, V. S. & BONAVIDA, B. (1977) Detection of soluble tumor-associated antigens in serum of tumor-bearing rats and their immunological role *in vivo*. *Cancer Res.*, **37**, 3385.

SCHULTZ, D. R. & YUNIS, A. A. (1979) Tumor-associated antigen in human pancreatic cancer. *J. Natl Cancer Inst.*, **62**, 777.

SCHWARZACHER, H. G. & WOLF, U. (Eds) (1974) *Methods in Human Cytogenetics*. Berlin: Springer-Verlag.

SJÖGREN, H. O., HELLSTRÖM, I., BANSAL, S. C. & HELLSTRÖM, K. E. (1971) Suggestive evidence that the "blocking antibodies" and tumour bearing individuals may be antigen-antibody complexes. *Proc. Natl Acad. Sci.*, **68**, 1372.

SPRENT, J. & MILLER, J. F. A. P. (1972) Thoracic duct lymphocytes from nude mice: Migratory properties and life-span. *Eur. J. Immunol.*, **2**, 384.

STERN, P. L., WILLISON, K. R., LENNOX, E. & 5 others (1978) Monoclonal antibodies as probes for differentiation and tumor-associated antigens: A Forssman specificity on teratocarcinoma stem cells. *Cell*, **14**, 775.

VON KLEIST, S., CHANY, E., BURTIN, P., KING, M. & FOGH, J. (1975) Immunohistology of the antigenic pattern of a continuous cell line from a human colon tumor. *J. Natl Cancer Inst.*, **55**, 555.

YOUNG, R. K., CAILLEAU, R. M., MACKAY, B. & REEVES, W. J. (1974) Establishment of epithelial cell line MDA-MB-157 from metastatic pleural effusion of human breast carcinoma. *In Vitro*, **9**, 239.


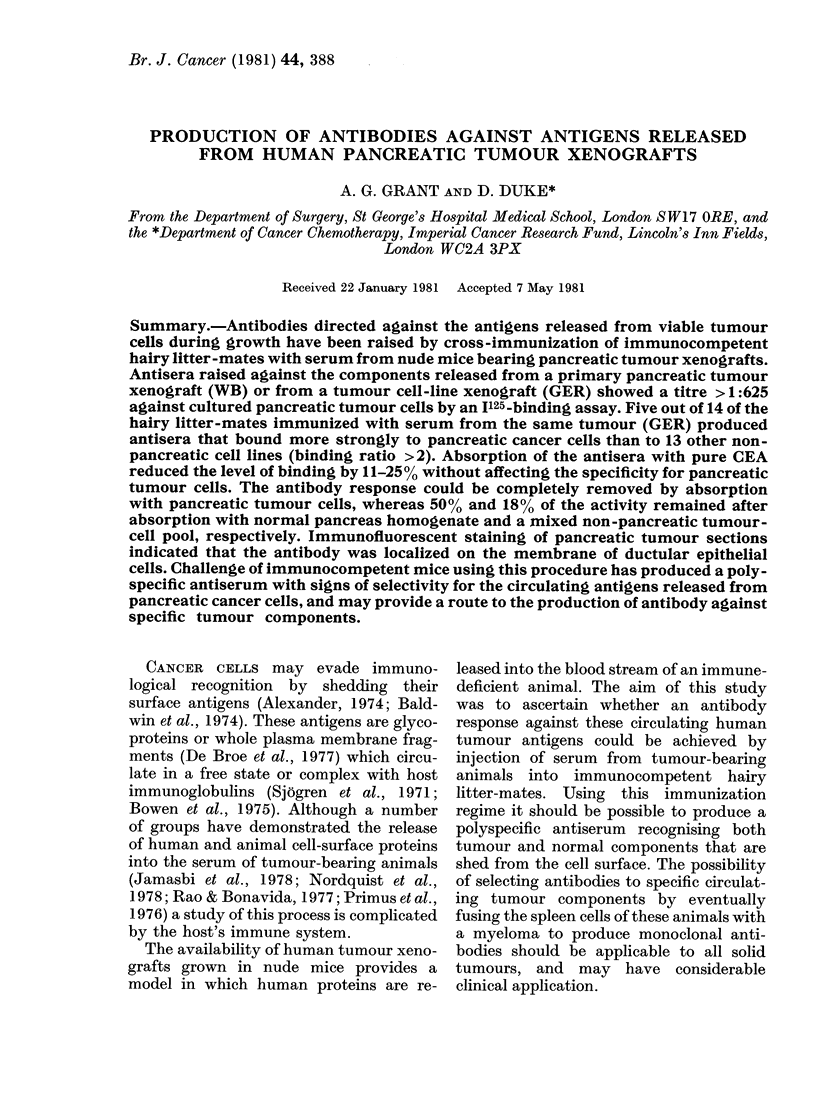

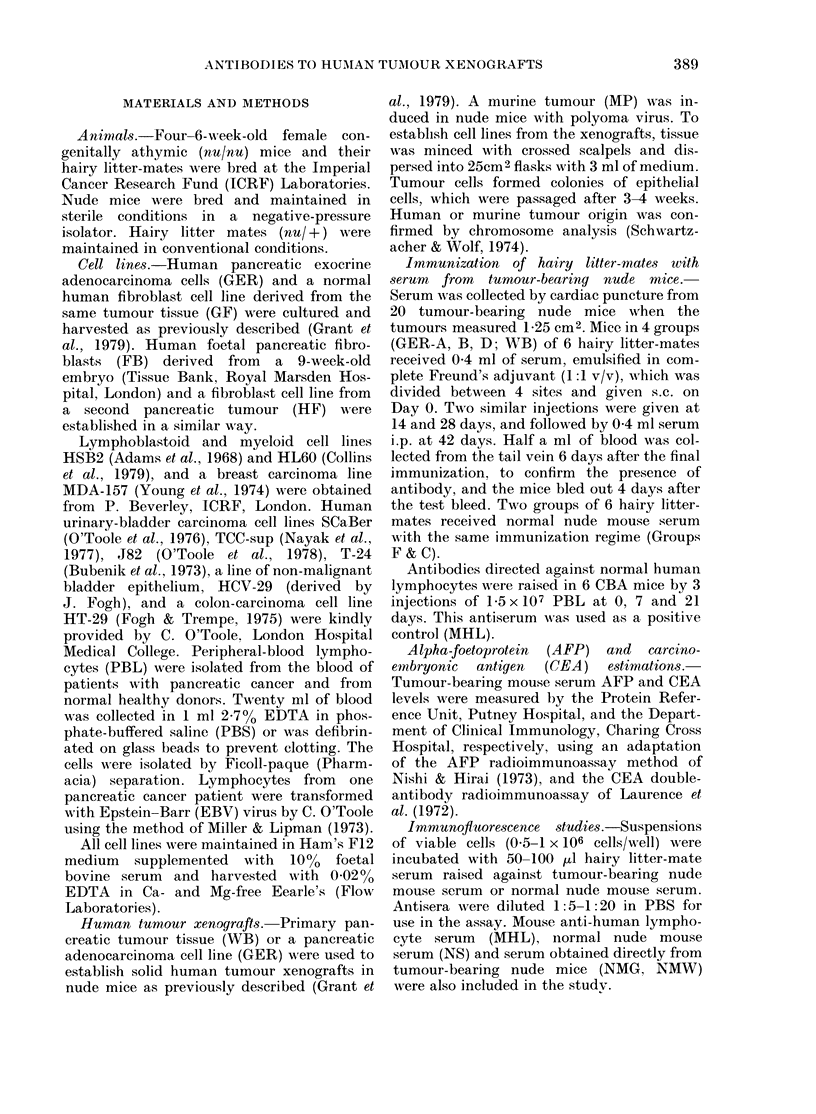

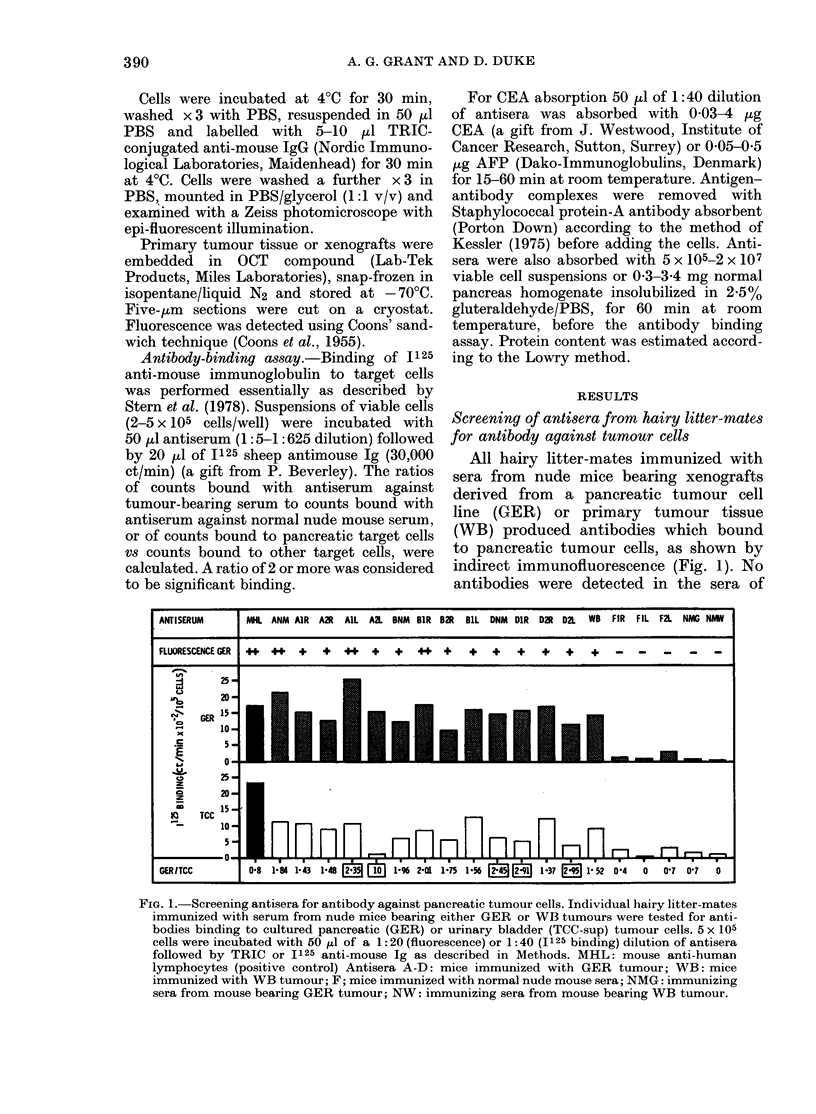

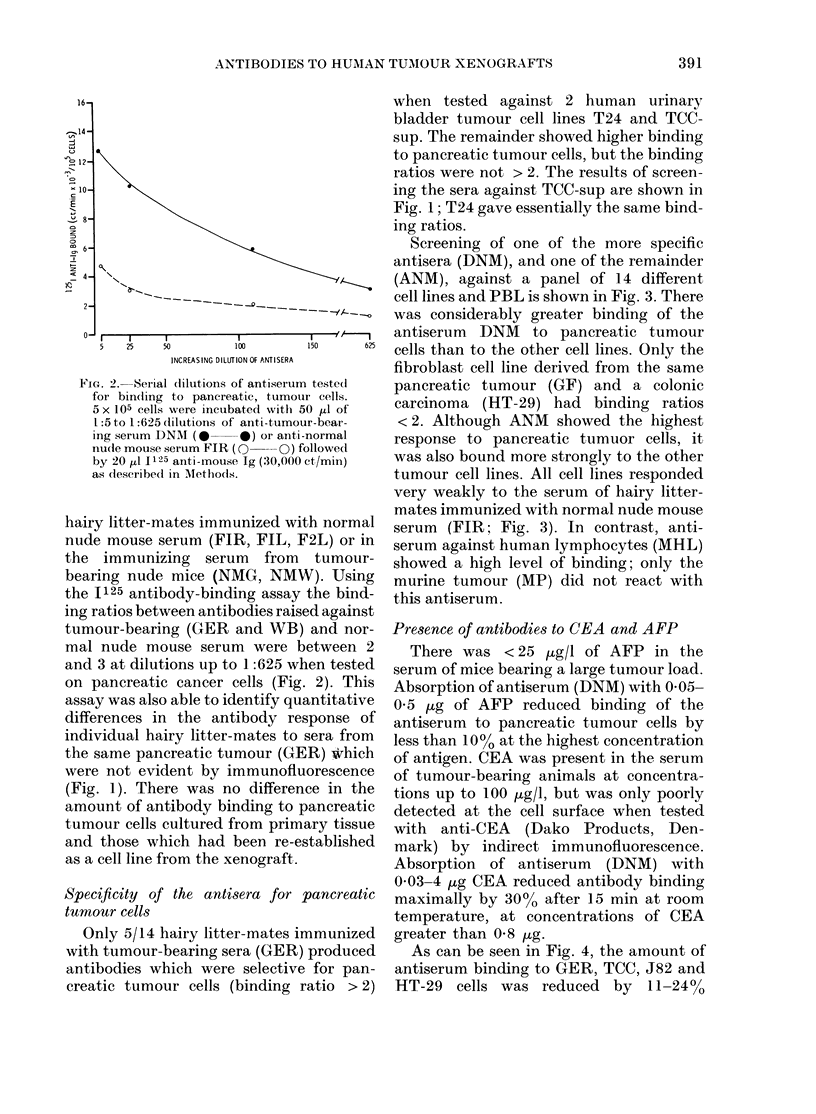

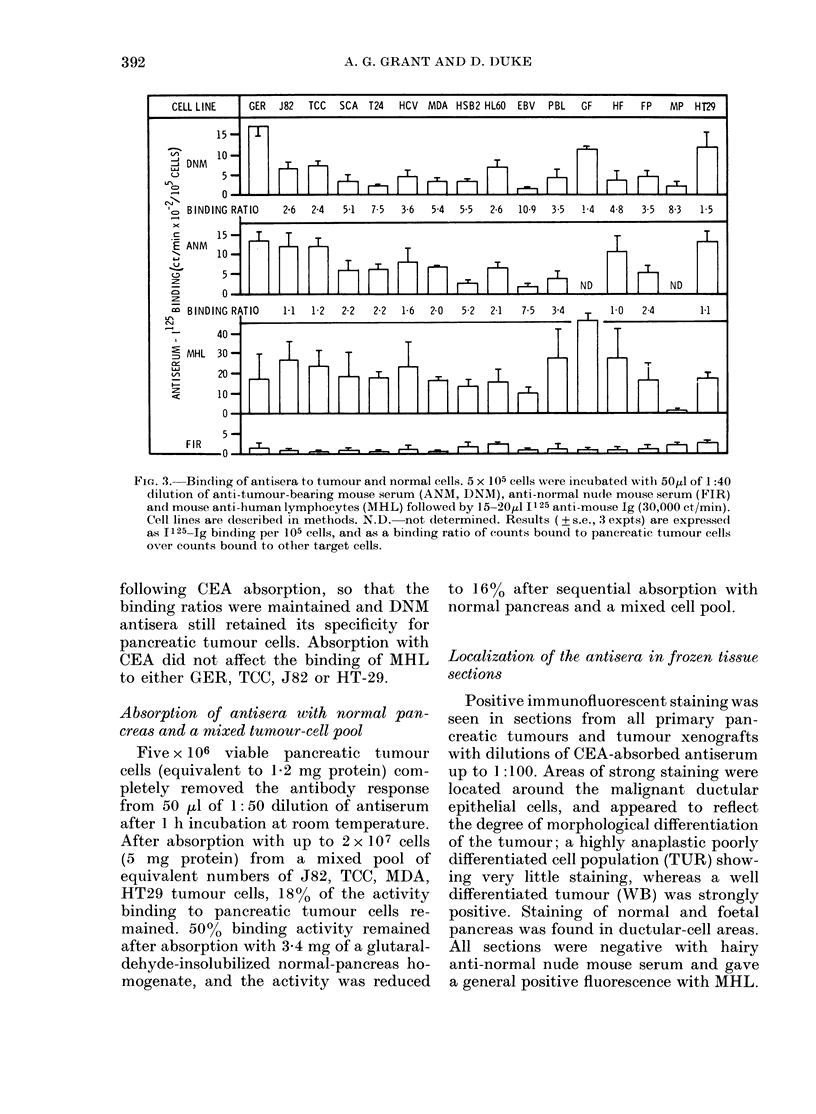

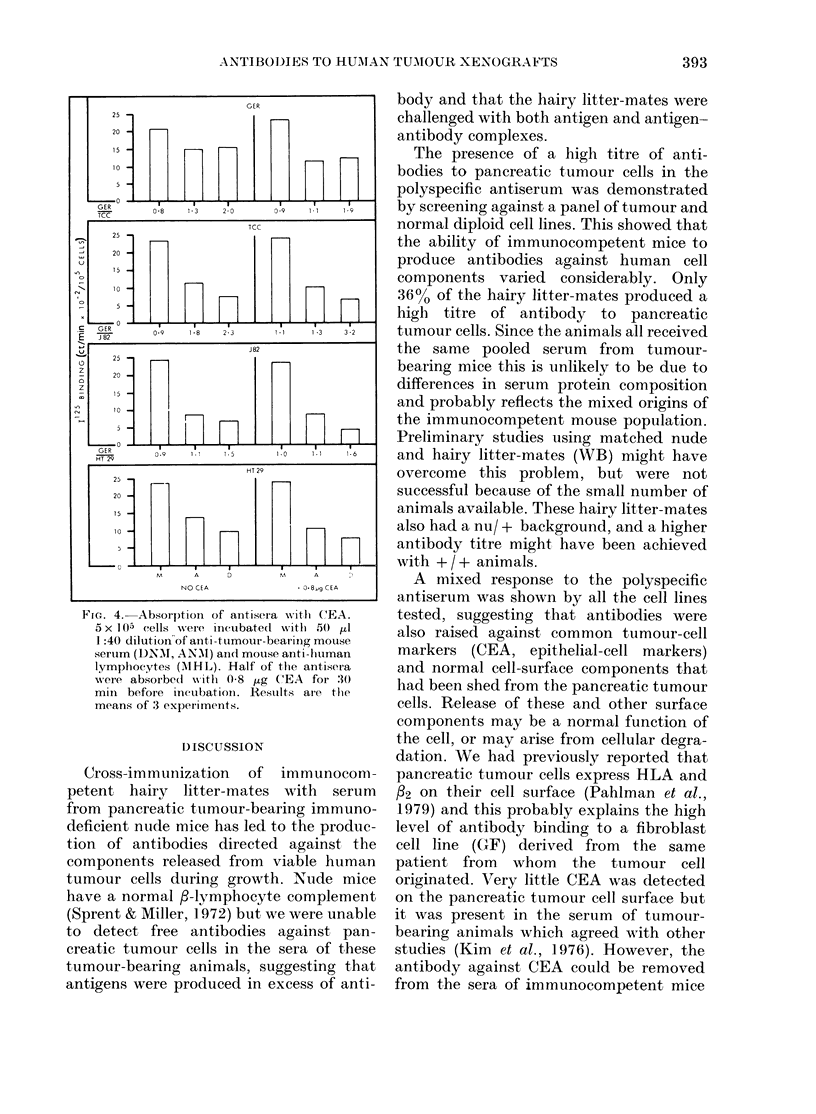

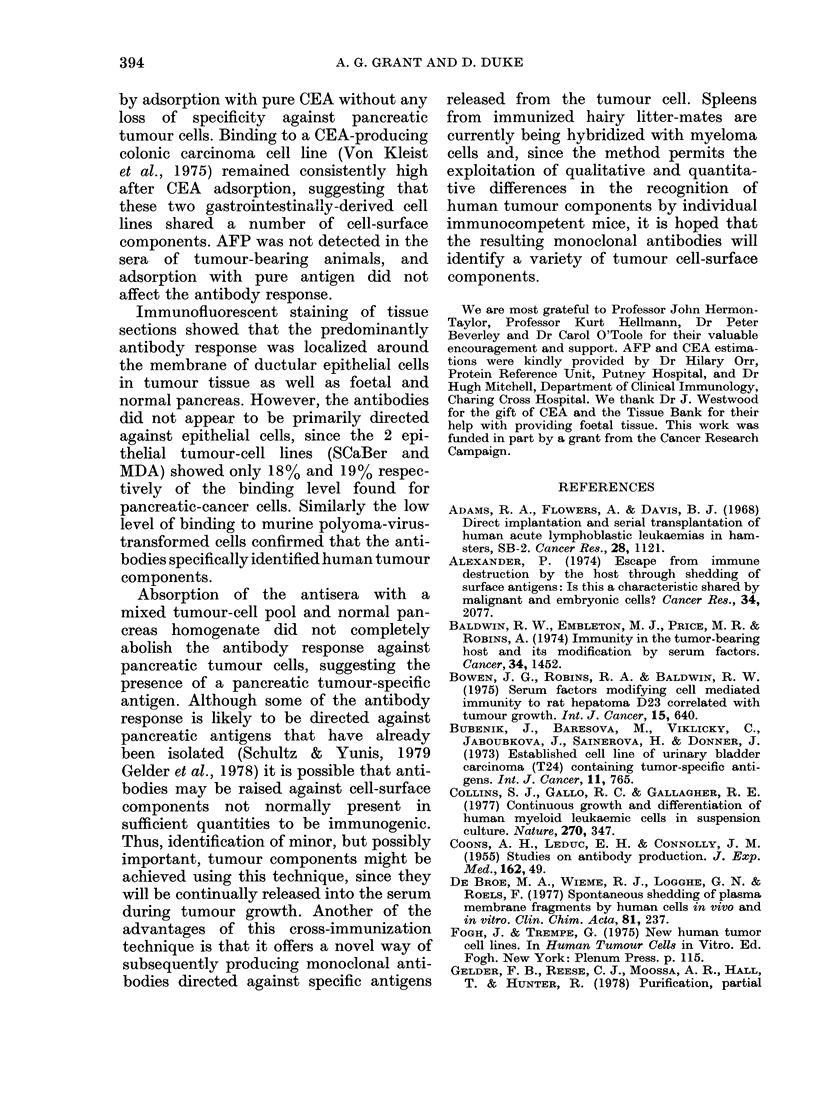

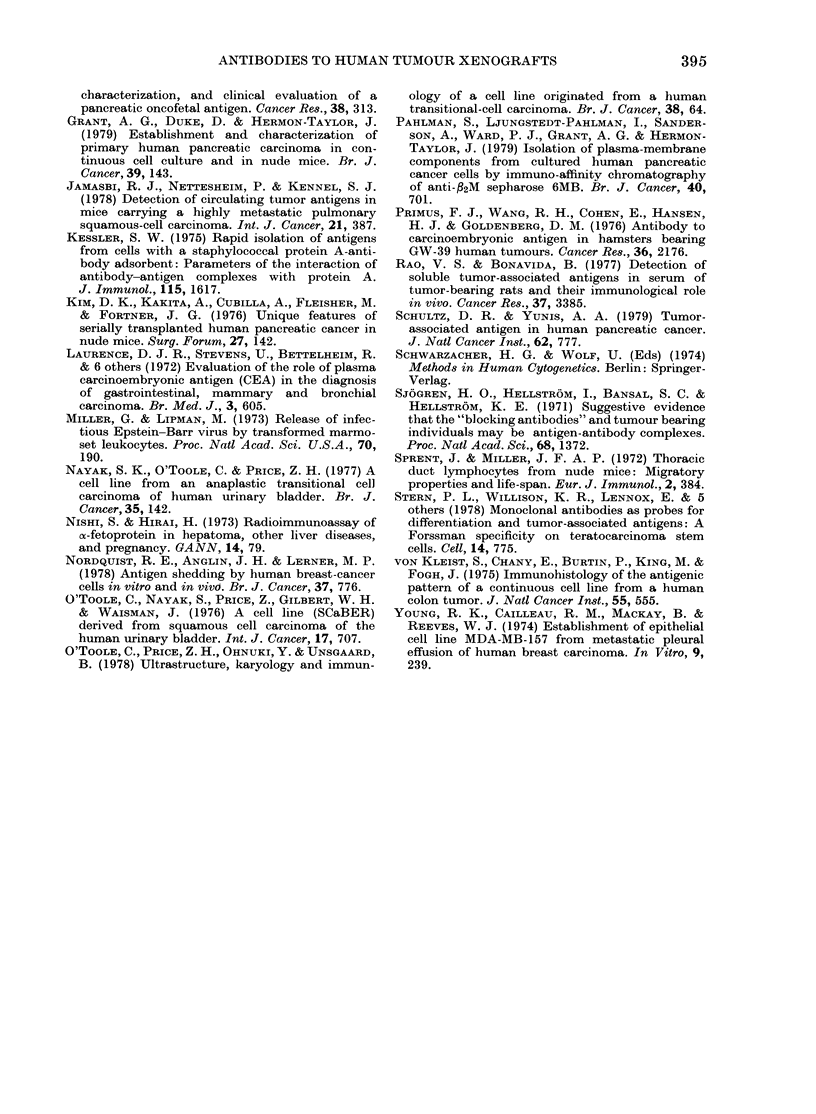


## References

[OCR_00665] Adams R. A., Flowers A., Davis B. J. (1968). Direct implantation and serial transplantation of human acute lymphoblastic leukemia in hamsters, SB-2.. Cancer Res.

[OCR_00671] Alexander P. (1974). Proceedings: Escape from immune destruction by the host through shedding of surface antigens: is this a characteristic shared by malignant and embryonic cells?. Cancer Res.

[OCR_00678] Baldwin R. W., Embleton M. J., Price M. R., Robins A. (1974). Immunity in the tumor-bearing host and its modification by serum factors.. Cancer.

[OCR_00684] Bowen J. G., Robins R. A., Baldwin R. W. (1975). Serum factors modifying cell mediated immunity to rat hepatoma d23 correlated with tumour growth.. Int J Cancer.

[OCR_00690] Bubeník J., Baresová M., Viklický V., Jakoubková J., Sainerová H., Donner J. (1973). Established cell line of urinary bladder carcinoma (T24) containing tumour-specific antigen.. Int J Cancer.

[OCR_00703] COONS A. H., LEDUC E. H., CONNOLLY J. M. (1955). Studies on antibody production. I. A method for the histochemical demonstration of specific antibody and its application to a study of the hyperimmune rabbit.. J Exp Med.

[OCR_00697] Collins S. J., Gallo R. C., Gallagher R. E. (1977). Continuous growth and differentiation of human myeloid leukaemic cells in suspension culture.. Nature.

[OCR_00708] De Broe M. E., Wieme R. J., Logghe G. N., Roels F. (1977). Spontaneous shedding of plasma membrane fragments by human cells in vivo and in vitro.. Clin Chim Acta.

[OCR_00727] Grant A. G., Duke D., Hermon-Taylor J. (1979). Establishment and characterization of primary human pancreatic carcinoma in continuous cell culture and in nude mice.. Br J Cancer.

[OCR_00734] Jamasbi R. J., Nettesheim P., Kennel S. J. (1978). Detection of circulating tumor antigens in mice carrying a highly metastatic pulmonary squamous--cell carcinoma.. Int J Cancer.

[OCR_00739] Kessler S. W. (1975). Rapid isolation of antigens from cells with a staphylococcal protein A-antibody adsorbent: parameters of the interaction of antibody-antigen complexes with protein A.. J Immunol.

[OCR_00746] Kim D. K., Kakita A., Cubilla A., Fleisher M., Fortner J. G. (1976). Unique features of serially transplanted human pancreatic cancer in nude mice.. Surg Forum.

[OCR_00752] Laurence D. J., Stevens U., Bettelheim R., Darcy D., Leese C., Turberville C., Alexander P., Johns E. W., Neville A. M. (1972). Role of plasma carcinoembryonic antigen in diagnosis of gastrointestinal, mammary, and bronchial carcinoma.. Br Med J.

[OCR_00759] Miller G., Lipman M. (1973). Release of infectious Epstein-Barr virus by transformed marmoset leukocytes.. Proc Natl Acad Sci U S A.

[OCR_00765] Nayak S. K., O'Toole C., Price Z. H. (1977). A cell line from an anaplastic transitional cell carcinoma of human urinary bladder.. Br J Cancer.

[OCR_00776] Nordquist R. E., Anglin J. H., Lerner M. P. (1978). Antigen shedding by human breast-cancer cells in vitro and in vivo.. Br J Cancer.

[OCR_00781] O'Toole C., Nayak S., Price Z., Gilbert W. H., Waisman J. (1976). A cell line (SCABER) derived from squamous cell carcinoma of the human urinary bladder.. Int J Cancer.

[OCR_00787] O'Toole C., Price Z. H., Ohnuki Y., Unsgaard B. (1978). Ultrastructure, karyology and immunology of a cell line originated from a human transitional-cell carcinoma.. Br J Cancer.

[OCR_00803] Primus F. J., Wang R. H., Cohen E., Hansen H. J., Goldenberg D. M. (1976). Antibody to carcinoembryonic antigen in hamsters bearing GW-39 human tumors.. Cancer Res.

[OCR_00797] Påhlman S., Ljungstedt-Poahlman I., Sanderson A., Ward P. J., Grant A., Hermon-Taylor J. (1979). Isolation of plasma-membrane components from cultured human pancreatic cancer cells by immuno-affinity chromatography of anti-beta 2M sepharose 6MB.. Br J Cancer.

[OCR_00815] Schultz D. R., Yunis A. A. (1979). Tumor-associated antigen in human pancreatic cancer.. J Natl Cancer Inst.

[OCR_00825] Sjögren H. O., Hellström I., Bansal S. C., Hellström K. E. (1971). Suggestive evidence that the "blocking antibodies" of tumor-bearing individuals may be antigen--antibody complexes.. Proc Natl Acad Sci U S A.

[OCR_00832] Sprent J., Miller J. F. (1972). Thoracic duct lymphocytes from nude mice: migratory properties and life-span.. Eur J Immunol.

[OCR_00809] Srinivasa Rao V., Bonavida B. (1977). Detection of soluble tumor-associated antigens in serum of tumor-bearing rats and their immunological role in vivo.. Cancer Res.

[OCR_00837] Stern P. L., Willison K. R., Lennox E., Galfrè G., Milstein C., Secher D., Ziegler A. (1978). Monoclonal antibodies as probes for differentiation and tumor-associated antigens: a Forssman specificity on teratocarcinoma stem cells.. Cell.

[OCR_00851] Young R. K., Cailleau R. M., Mackay B., Reeves W. J. (1974). Establishment of epithelial cell line MDA-MB-157 from metastatic pleural effusion of human breast carcinoma.. In Vitro.

[OCR_00845] von Kleist S., Chany E., Burtin P., King M., Fogh J. (1975). Immunohistology of the antigenic pattern of a continuous cell line from a human colon tumor.. J Natl Cancer Inst.

